# Authors’ Response to Peer Reviews of “The Role of Anxiety and Prosocial Behaviors on Adherence Behaviors to Prevent COVID-19 in University Students in the United States: Cross-Sectional Study”

**DOI:** 10.2196/58859

**Published:** 2024-05-27

**Authors:** Silvia Corbera, Amanda M Marín-Chollom

**Affiliations:** 1 Department of Psychological Science, Central Connecticut State University, New Britain, CT, United States

**Keywords:** prosocial behavior, COVID-19, anxiety, COVID-19 prevention, preventive health behavior, adherence to prevention

*This is the authors’ response to peer-review reports for “The Role of Anxiety and Prosocial Behaviors on Adherence Behaviors to Prevent COVID-19 in University Students in the United States: Cross-Sectional Study.”*

## Round 1 Review

### Summary Provided by Reviewers [1]

*The study [* 2 *] investigates the complex interplay between anxiety (both state and trait), prosocial behaviors, and adherence to COVID-19 preventive measures among college students. While overall prosocial behaviors did not directly correlate with anxiety, a seemingly significant crossover effect emerged in relation to public prosocial behaviors, suggesting that individuals with lower self-oriented tendencies exhibited increased adherence behaviors under heightened state anxiety. The study used a quantitative research design with a sample of 54 undergraduate students, using online questionnaires to measure various psychological factors and preventive behaviors. Individuals with high anxiety showed increased adherence to preventive measures, contrary to the hypothesized moderating effect of prosocial behaviors. Overall, the reviewers appreciated the effort and recognized the challenges of conducting a research study in the context of an unprecedented social condition. However, the findings are challenged by weak effect sizes, multiple comparisons, and unclear appropriateness of using prosocial behaviors as a moderating variable. The study underscores the psychological impact of the COVID-19 pandemic on college students and suggests the need for further exploration into the nuanced relationships between anxiety, prosocial behaviors, and adherence to public health guidelines. Despite its strengths in data collection and questionnaire use, limitations such as a narrow participant pool and reliance on self-reporting warrant cautious interpretation of the results. The study encourages future research to delve deeper into these intricate connections, offering insights into potential interventions for promoting adherence to COVID-19 preventive measures and beyond.*

Response: We thank the editor and reviewers for their valuable time and insightful and helpful comments. In this resubmission, we have provided answers to your comments below and improved the manuscript following your contributions.

*Below we list major and minor concerns that were discussed by participants of the live review, and where possible, we provide suggestions on how to address those issues.*

### List of Major Concerns and Feedback

#### Small Sample Size and Mediation Analysis

*One of the main concerns raised in the discussion was the small number of study participants. This is acknowledged as a limitation factor in the discussion, but what is less clear is if a mediation analysis is the right approach to analyze these data. One reviewer suggested the use of a multivariate analysis instead as it would take all variables into account without forcing potentially artificially generated causal mediations between variables that don’t show an obvious causal dependency. Another reviewer, however, felt that while this approach may work and it would be useful to explore, using a multivariate analysis may lead to overfitting in most covariates given the small sample size and given that 86% of subjects reported anxiety. Overall, the suggestion is for the authors to provide a rationale for selecting anxiety as a mediator variable over prosocial tendencies, or vice versa, and possibly explore other analyses and comment on the limitations of the approaches.*

Response: We thank the reviewers for this comment. We wanted to clarify that this study used a moderation analysis, not a mediation analysis. The goal was not to examine causality but the moderating effects of prosocial tendencies in the relationship between anxiety and adherence to preventive behaviors to prevent the spread of COVID-19. Given that the study was conducted during the second wave of the pandemic, the reported stress levels of the undergraduate students who participated in the study were high (86% of subjects reported anxiety); the goal of the study was to examine the relationship between stress levels and adherence to preventive behaviors to prevent the spread of COVID-19, and if this relationship was moderated by prosocial tendencies. Based on the scientific literature, which robustly indicates that stress influences decision-making processes [3,4], we wanted to examine if this finding would be replicated instead of using laboratory stress-inducing techniques, using the COVID-19 pandemic as the natural worldwide stressor and the decision-making process of adhering to preventive behaviors. However, the scientific literature is scarce in regard to the role of prosocial behaviors in situations of stress, and therefore, we were particularly interested in examining the role of prosocial behaviors in individuals who would show high versus low prosocial behaviors.

In regard to the small sample size, we performed a power analysis using SPSS with a power (β) set at 0.8, the Cohen *f* at 0.15 for medium effects, and significance level at .05. The sample needed was 55. Therefore, we consider that the study was sufficiently powered as our study had 54 participants. We acknowledge that the sample size was small, but the study was completed during a second wave of COVID-19, with a limited number of participants at the time. We were particularly interested in examining the role of anxiety at that moment, given the exceptionality of the situation. Worldwide life circumstances changed greatly after that spring (with full availability of the vaccines to everyone including young adults) and have changed now, and the study’s historical environment is different from what we captured, so it was not possible for us to add more participants to this study. We have now acknowledged this further in the Limitation section of the manuscript.

#### Uniform/Convenience Sampling

*The reviewers acknowledged the study’s challenging circumstances and the effort to capture unique data. However, they expressed concern about generalizability, noting that all participants were undergraduates from one college. The demographic homogeneity of this group may limit applicability to diverse populations, raising caution about extrapolating results across age groups, educational backgrounds, and cultural contexts. The convenience sampling method may have introduced bias, as easily accessible participants might not represent the broader target population.*

Response: We thank the reviewers for this insightful comment. We acknowledged in the Limitation section that the sampling method used was convenience sampling with an undergraduate college population. We agree with the reviewers that this homogeneous sample may limit the applicability to diverse populations, and therefore, we now further clarified this limitation as suggested by the reviewers by raising caution about extrapolating results across age groups, educational backgrounds, and cultural contexts. At the same time, we wanted to point out the idiosyncratic developmental challenges of the undergraduate population, as for most of them, the college experience is already a significant change in their life, let alone in the middle of a worldwide pandemic. Research has shown that the undergraduate population was a specific vulnerable population affected greatly by the stressors of the COVID-19 pandemic, especially among the lower socioeconomic undergraduate groups [5,6]. Therefore, even though the sample was not representative of other age groups, we consider that it merits study. We have now further clarified this item in the Limitation section.

#### University Policy and Mask Mandates

*The study doesn’t explicitly consider the potential impact of university campus policies during the pandemic, such as mask mandates and social distancing, on adherence behaviors. The findings may be influenced by the specific characteristics of the chosen university, including its restriction policies. For example, students on campus may wear face masks due to safety requirements when entering campus common spaces versus through their own personal decision process. Therefore, exercising caution in generalizing conclusions to broader contexts is suggested. Reviewers highlight the importance of future research with more diverse samples to enhance external validity, reinforcing the study’s overall robustness. This provides a constructive pathway for refining the study’s scope and applicability.*

Response: We thank the reviewers for this comment. We stated in response to the “Uniform/Convenience Sampling” comment that we added further information in the Limitation section regarding the caution of extrapolating the results to other populations and contexts. We agree that the university policies at the time were exceptional. However, we wanted to note that the measure that we used to examine the frequency of engaging in COVID-19 preventive behaviors asked questions that were aimed at all aspects of their life not specific to any context, and in addition, they also had the option to answer “I don’t know/prefer not to answer/Not applicable” for each question. Therefore, participants had the option of not answering and not answering because the question was not applicable to their life such as “Avoiding Playdates (letting children play with other children).” Specifically, the questionnaire asked “Please indicate the frequency with which you have adopted each action/behavior in the previous 7 days:” in which they had to answer items such as “Hand washing with soap and water,” “Using a hand sanitizer,” “Wearing a Face Mask,” “Avoiding non-essential travel,” and “Avoiding opening the mail or delivered goods” using a 5-point scale (“Most of the time,” “Some of the time,” “Seldom,” “Never,” and “I don’t know/prefer not to answer/Not applicable”). At the time of the pandemic in the spring of 2021, the safety measures applied by the university were many, including social distancing and mask wearing, but also followed state-mandated measures as the state had a travel advisory and a requirement to complete a travel health form and quarantine for a 10-day period if traveling outside of Connecticut for more than 24 hours in states other than New York, New Jersey, and Rhode Island or countries other than the United States. Therefore, this mandate was statewide. Additionally, at the time, spring 2021, vaccinations were provided for priority populations such as those older than 75 years, and vaccinations for those 65 years and older were not planned until mid-February. Vaccinations for everyone 16 years and older were not available until April 1. Additionally, there was a ban for in-person research during the spring of 2021 that was not expected to be lifted until May 2021, which is when the semester was over. We believe that the situation was exceptional on campus and outside campus, and the measures on campus were designed for safety. Additionally, students had the option of not answering the items of the questionnaire; therefore, we believe that the likelihood that the university policies influenced their responses to be biased in any way was very small.

*The study’s cross-sectional design and reliance on self-reporting introduce potential limitations in establishing causal relationships and accurate data collection. (In general, no retrospective exploratory study can show causality, asserting a causal relationship amounts to the post hoc ergo propter hoc logical fallacy.) The one-time nature of the study also limits insights into the dynamic nature of psychological factors and preventive behaviors over time. Caution should be exercised in interpreting the results, as correlation does not imply causation.*

Response: We thank the reviewers for this comment, and we added this in the Limitation section. In addition, in our Discussion section, when interpreting the results, we did not imply causation in any instance, as we were carefully using the words “association” in lines 1 and 2 of the discussion: “The present study aimed to examine the association between state and trait anxiety during the COVID-19 pandemic and the adherence behaviors to prevent the spread of COVID-19...”; we also use the phrase “more likely to” such as in line 6 of the Discussion: “...we hypothesized that participants with high state and trait anxiety would be more likely to adhere...”; also, the word “relationship” was used in line 10: “...and that this relationship would be stronger for individuals with high prosocial behavior tendencies....” Therefore, we were careful to not imply causation. We changed the wording in line 33 of the Discussion to ensure causality was not implied.

*Furthermore, reviewers don’t think that the results of this study can be used on their own to make any definitive public health policy recommendations.*

Response: We agree and we acknowledge this in the Limitation section but can inform other future studies on the role of prosocial behaviors in the relationship between stress and adherence to preventive behaviors. We added the following statement: “Fourthly, because of the small sample size, the cross-sectional nature of the study, this results on their own cannot make any definitive public health policy recommendations but can inform other studies on the role of prosocial tendencies in the relationship between stress and adherence to preventive behaviors.”

#### Adherence Scores

*The study mentions adherence to COVID-19 public health safety recommendations as an outcome variable. There is a need for more clarity on how the adherence scores were calculated, especially considering potential confounding factors such as university campus policies during the pandemic.*

Response: We thank the reviewers for these comments. We provided information regarding the influence of “campus policies during the pandemic” in the answer to the “University Policy and Mask Mandates” comment. Also, regarding the calculation of the adherence scores to COVID-19 preventive behaviors, we described the calculation in the Methods section. Please see below the wording from the Methods section:

“COVID-19 Preventative Behaviors. The COVID-19 International Survey (CIS) from the PhenX Toolkit (2020) was used to collect data on what preventative COVID-19 behaviors participants were engaging in. The 23 items within the survey were specific to what frequency individuals are engaging in COVID-19 preventative behaviors were used for this study (eg, hand washing, mask wearing physical distancing, avoiding social gatherings, self-quarantining after travel, self-quarantining if infected or likely infected were used). These items are answered on a 4-point Likert scale from never to most of the time, with the additional option of ‘don’t know/I prefer not to answer/Not applicable.’ One total sum score was calculated, and higher scores indicated higher engagement in COVID-19 preventative behaviors.”

#### Missing Data

*The study’s approach to handling missing data in nonmandatory survey questions is not explicitly discussed. This may impact the results and should be clarified.*

Response: We thank the reviewers for this insightful comment. Most participants had complete data for the variables used in the analysis, except for age (n=53) and trait anxiety (n=44). State anxiety had complete data (N=54). The lack of effects with trait anxiety could be due to the smaller sample size, and state anxiety may be more relevant to the COVID-19 pandemic context. We have now added this information in the Discussion section.

#### Reliability Metrics

*The study does not provide information on test-retest reliability, accuracy against a gold standard, or error of measurement for the Prosocial Tendencies Measure (PTM) reliability. Reliability induction from other studies is mentioned, but the study population’s specific reliability is not demonstrated. Without these critical reliability metrics, the study leaves a gap in the assessment of the psychometric properties of the PTM. Including such information would enhance the transparency and credibility of the study’s findings, allowing readers to better evaluate the reliability and validity of the instrument used to assess prosocial behaviors. Future research may consider providing a comprehensive assessment of the psychometric properties of measurement instruments to strengthen the methodological rigor and overall quality of the study.*

Response: We thank the reviewers for this comment. We have now added the validity and reliability information of this measure in the Methods section of the manuscript.

#### Statistical Model and Data Selection

*Some reviewers expressed concern related to the lack of transparency about how variables were selected as moderators or mediators, how some others (eg, age) were chosen to be excluded, and how others were chosen to be reported on from the cited “larger study.” Adding clarity around the rationale that led to making such choices would help the reader better contextualize the results.*

Response: We thank the reviewers for this comment. In this study, we used a moderation analysis, not a mediator analysis. The variables were chosen based on the literature and theory that drove the research protocol. In our institutional review board (IRB) protocol (#20089), we proposed testing two models, and these results are from one of those models driven by the literature.

*Furthermore, a scoring guide for the CIS Survey would be helpful to add. There is a concern that a simple sum method may be biased because some questions may not be relevant to all subjects (eg, playdates only impact subjects that have childcare responsibilities).*

Response: We thank the reviewers for this suggestion. We provided information on this scale in the Methods section and in response to the “University Policy and Mask Mandates” and “Adherence Scores” comments. We want to emphasize that participants had the option to answer “I don’t know/prefer not to answer/Not applicable” for each question. Therefore, participants had the option of not answering if it was not applicable to them, such as “Avoiding Playdates (letting children play with other children).”

#### Ethics

*While the study mentions obtaining IRB approval and online passive consent, specific details regarding confidentiality, privacy safeguards, and participant understanding of risks are not thoroughly addressed.*

Response: We agree and we disclosed that this study was fully IRB approved. At Central Connecticut State University, studies that are IRB approved are shown to follow proper confidentiality procedures and privacy as well. All these procedures were followed in this study, as per the IRB approval obtained in this IRB protocol (#20089).

*Furthermore, it is not clear what the authors mean by “passive consent.”*

Response: We agree and we understand the confusion, and we removed the word passive consent and added the following wording: “Participants were presented with an online informed consent, and they acknowledged it by pressing a button to continue the study.”

#### Data and Reproducibility

*The study provides a moderate level of detail, but more specific information is needed for reproducibility. This includes additional demographic details, exact questionnaire wording, and more details on moderation analyses. The study would benefit from providing a more comprehensive set of demographic information about the participants such as age distribution, gender distribution, and other relevant characteristics. A richer demographic profile would contribute to a more nuanced understanding of the study population and facilitate comparisons with other research. Reviewers suggested adding available details to Table 1.*

Response: We thank the reviewers for this insightful comment. We now added additional demographic details in the manuscript describing the participants further. As described now in the manuscript, we added the age range and percentiles, and we added a full table—a new Table 1 that includes a description of the demographic variables: gender, race/ethnicity, enrollment status, whether they are first generation students, marital status, employment status, hours of work per week, and housing situation and living situation.

*While the study mentions that data are available upon reasonable request, reviewers suggest considering providing additional information on how interested researchers can request the data, perhaps from the corresponding author or another designated contact. This could enhance transparency and facilitate potential collaborations or further scrutiny of the results.*

Response: We agree with the reviewers on this comment, and we now added in the Data Availability statement that “the data generated from this study can be available from the corresponding author upon reasonable request.”

### List of Minor Concerns and Feedback

#### Readability

*Overall, the reviewers thought that the manuscript would benefit from a clearer explanation of key terms and recommended keeping the terminology consistent across the manuscript so as to help the reader better follow the narrative and interpret the findings. For example, there was some confusion among reviewers on the meaning of “public prosocial scale.”*

Response: We thank the reviewers for this comment, and we now have added extensive clarifications in the Discussion section to enhance the readability of the manuscript and concretely remove any possible confusion with the definition of the public prosocial scale.

#### Approach and Results

*It may be helpful to show more information about some of the background variables. One question is if the deviation of age from a normal distribution is significant and thus a possible contributor to the study’s findings if age correlates with adherence or anxiety. Showing not only mean and SD but also median, quartiles, and range may provide a better feel for what the study population, or at least the participant sample, is like.*

Response: We thank the reviewers for this comment. We had already included the mean and SD of age, and we now added the range and the quartiles. Additionally, we now provide an extensive list of demographic variables in Table 1. As the reviewers can see, the sample did not have much variability in age as they were all undergraduate students, and we did not expect that the age differences would be a differential contributor to the study findings. In response to the reviewers, we calculated the correlation between age and state and trait anxiety and adherence, and none of the results were significant (trait anxiety: *r*=0.109; *P*=.48; state anxiety: *r*=0.125; *P*=.37; adherence: *r*=−0.015; *P*=.91).

*It may be useful to make explicit the assumptions underlying the modeling and parameters used for PROCESS, such as the degree of independence of the moderator.*

Response: The parameters for using moderation in PROCESS are like any moderation analysis program. The only difference is the bootstrapping, which is specified in the Methods section. We now added this information in the manuscript and the new reference as well.

Hayes AF. *Introduction to Mediation, Moderation, and Conditional Process Analysis.* 3rd Edition. Guilford Press, 2022.

#### Discussion

*The authors may consider adding a section to the discussion to explore variables related to vaccine hesitancy and other factors (eg, sense of invincibility) as a suggestion for future research, expanding the scope beyond adherence to preventive measures.*

Response: We agree with this comment, and we expanded on this topic with additional sentences added in the Discussion section.

*Given the reliance on self-report measures, the reviewers suggest the authors discuss the potential impact of social desirability bias on participants’ responses. Addressing this concern would add transparency to the limitations of the study.*

Response: We agree with this comment. We wanted to point out that we had already addressed this issue in the Limitation section.

*Reviewers suggest authors discuss how the results support following up further on correlations among PTM scales and on the possible moderator effect of public prosocial tendencies, with recommendations for including a broader set (explicitly listed) of potentially explanatory independent variables.*

Response: We agree with the reviewer’s comments. We now added a list of potential variables that may have also influenced the results in the Discussion section. We added the following paragraph: “Additionally, other variables that may have influenced the results such as vaccine hesitancy, perceived personal risk and disease vulnerability, and trust in science may be potential variables to study in future research, especially regarding the factors that may impact the adherence to preventive behaviors in young adults [7,8].”

*It may also be helpful to add some explanation of why the psychometric characteristics of the survey instruments as established in other studies can be trusted to be the same as used in this study (online, unsupervised, etc). Some reviewers found it concerning that this study found statistically significant pairwise associations between PTM subscales, and this should be addressed, perhaps with speculation about why this happened.*

Response: We thank the reviewers for this insightful comment. We now added the validity and reliability information of all the PTM subscales and of the State-Trait Anxiety Inventory. We also further elaborated on the possible reasoning for the differential results of the PTM in the Discussion section.

#### Figures and Tables

*Consider using a 2 × 2 table in Figure 1 to illustrate the detected moderator effect.*

Response: We thank the reviewers for this comment. However, because of the many tables we already have in the study and the fact that we added an additional one with the demographics (Table 1), we decided to use this figure that illustrates the study’s effects without having to have 2 figures.

#### Title

*Given the concern about generalizability, a reviewer suggested the authors consider changing the title to “Adherence Behaviors to Prevent COVID: The Role of Anxiety and Prosocial Behaviors Amongst University Students in the US.”*

Response: We thank the reviewers for this comment, and we also considered that it would be important to add the dates of the study to describe the historical circumstances; therefore, we also added dates to increase awareness of the time period. Therefore, the manuscript is now titled “Adherence Behaviors to Prevent COVID: The Role of Anxiety and Prosocial Behaviors Amongst University Students in the US- January 2021-May 2021.”

## Abbreviations

IRB: institutional review board
PTM: Prosocial Tendencies Measure

## References

[1] Arogundade FQ, Azra S, Pulier M, Ram L, Saderi D, Tomaskova M (2024). Peer review of “The Role of Anxiety and Prosocial Behaviors on Adherence Behaviors to Prevent COVID-19 in University Students in the United States: Cross-Sectional Study". JMIRx Med.

[2] Corbera S, Marin-Chollom AM (2024). The role of anxiety and prosocial behaviors on adherence behaviors to prevent COVID-19 in university students in the United States: cross-sectional study. JMIRx Med.

[3] Starcke K, Brand M (2012). Decision making under stress: a selective review. Neurosci Biobehav Rev.

[4] Nowacki J, Heekeren HR, Deuter CE (2019). Decision making in response to physiological and combined physiological and psychosocial stress. Behav Neurosci.

[5] Rudenstine S, McNeal K, Schulder T (2021). Depression and anxiety during the COVID-19 pandemic in an urban, low-income public university sample. J Trauma Stress.

[6] Lee J, Solomon M, Stead T, Kwon B, Ganti L (2021). Impact of COVID-19 on the mental health of US college students. BMC Psychol.

[7] Gupta S, Watanabe S, Laurent SM (2021). Psychological predictors of vaccination intentions among U.S. undergraduates and online panel workers during the 2020 COVID-19 pandemic. PLoS One.

[8] Hromatko I, Tonković M, Vranic A (2021). Trust in science, perceived vulnerability to disease, and adherence to pharmacological and non-pharmacological COVID-19 recommendations. Front Psychol.

